# Outlier-Resistant Initial Alignment of DVL-Aided SINS Using Mahalanobis Distance

**DOI:** 10.3390/s25247599

**Published:** 2025-12-15

**Authors:** Yidong Shen, Li Luo, Guoqing Wang, Tao Liu, Lin Luo, Jiaxi Guo, Shuangshuang Wang

**Affiliations:** 1 School of Electronic Information and Electrical Engineering, Chengdu University, Chengdu 610106, China; 202310303224@cdu.edu.cn (Y.S.); 202310302119@cdu.edu.cn (L.L.); 202310303102@cdu.edu.cn (J.G.); 2 School of Information and Control Engineering, China University of Mining and Technology, Xuzhou 221116, China; guoqingwang@cumt.edu.cn; 3 Chengdu Kinyea Technologies Company Ltd., Chengdu 610000, China; 4 School of Literature and Journalism, Chengdu University, Chengdu 610106, China; 202310611420@cdu.edu.cn

**Keywords:** robust initial alignment, SINS, Doppler velocity log, outlier detection

## Abstract

Due to the influence of the complex underwater environment, the initial alignment method for Doppler velocity log (DVL)-aided strap-down inertial navigation systems (SINS) often suffer from performance degradation, especially when DVL measurements are contaminated by outliers. In this paper, an outlier-resistant Initial Alignment method with interference suppression for SINS/DVL integrated navigation system is proposed, by which, by constructing an improved Mahalanobis distance anomalous detection criterion, the anomaly of the residual vector composed of observation vectors is judged, and an adaptive weighting factor is introduced into the observation matrix to suppress the abnormal interference in the alignment process. Simulation and experimental results show that, compared with existing initial alignment methods, the proposed method achieves higher alignment accuracy in the presence of outliers, which is more suitable for the SINS/DVL integrated navigation system.

## 1. Introduction

Strapdown inertial navigation systems (SINS) are widely applied in underwater vehicles due to their continuity and autonomy, while Doppler velocity logs (DVL) provide velocity measurements without error accumulation [1,2,3]. The integration of SINS and DVL has thus become a standard navigation scheme for underwater applications. As the prerequisite for integrated navigation, the alignment process critically influences the overall accuracy and stability [4,5]. Although conventional analytic coarse alignment methods [6,7] and self-alignment methods [8,9,10,11,12] are well developed for static or oscillatory bases, they fail under dynamic conditions. In particular, in-motion alignment techniques have attracted extensive research attention to mitigate maneuver-induced limitations [13,14,15,16].

Employing external information to assist SINS initialization has been recognized as an effective strategy to mitigate the impact of motion disturbances on alignment accuracy. In [17], Wu first introduced the optimization-based alignment (OBA) method, where observation vectors are derived from the integration of the specific force equation, and the initial attitude matrix is subsequently obtained using attitude determination methods, thereby enabling in-motion alignment. Building on this foundation, Wu further proposed the velocity-integration-based OBA and position-integration-based OBA methods [3], which enhanced the accuracy of observation vector computation. To alleviate error accumulation during the integration process, a sliding-window-vector construction-based OBA method was later developed. Various extensions of OBA have also been reported, including DVL-aided OBA [18], odometer-aided OBA [19,20], velocity-aided OBA [21], and backtracking scheme based OBA methods [22,23]. While these methods are computationally efficient and structurally concise, their theoretical foundation still relies on least-squares estimation, making them sensitive to measurement disturbances. In complex environments, external information is prone to outliers, which may degrade alignment accuracy or even cause alignment failure. Therefore, enhancing the robustness of in-motion alignment has become one of the key challenges currently faced in the navigation field.

To mitigate the impact of outlier disturbances in in-motion alignment, Xu proposed a robust Kalman filter–based method [24]. In this approach, an observation vector model is first derived under a gravity-motion framework, and robust Kalman filtering is then employed to detect and reject outliers in real time. The filtered estimates are subsequently used to reconstruct the observation vectors, thereby achieving a robust OBA solution. In [25,26], Xu further introduced a weight-matching OBA method inspired by Huber’s robust estimation theory [27]. This method incorporated the Huber function into the OBA framework, established a statistical model for outlier detection parameters, and demonstrated strong anti-interference capability. However, the key parameter of the Huber weighting mechanism is typically set to a fixed empirical value [28]. Such a static threshold lacks adaptability to dynamic variations in measurement statistics, which may lead to misclassification or suboptimal weighting under non-stationary noise conditions, thus limiting its adaptability and optimal performance in more complex environments.

To this end, this paper proposes an outlier-resistant initial alignment method based on a refined Mahalanobis distance criterion. The method first constructs a residual vector model using the original observation vectors within the OBA framework, and then recursively updates the Mahalanobis distance corresponding to the current observation vector. Subsequently, the mean and variance of Mahalanobis distances from historical epochs are utilized to dynamically establish an anomaly detection criterion. An exponential-type flexible weighting function is then designed to adaptively adjust the weights of the observation vectors according to the anomaly detection results, thereby suppressing the impact of outlier disturbances in the OBA process. The effectiveness of the proposed method is validated through both simulations and experimental data. The results demonstrate that the method ensures the stable convergence of attitude errors over time, even under outlier disturbances, and achieves higher alignment accuracy and better robustness compared with state-of-the-art methods. The contributions of paper are as follows:

- A detection method was presented under the OBA framework and recursively calculates the Mahalanobis distance. By using historical statistics to adaptively set the anomaly detection threshold, it achieves real-time and statistically meaningful effective identification of outliers in the observed sequence.
- An exponential-type adaptive weighting function based on the detection mechanism was imported to realize the dynamic adjustment of the contribution of each historical epoch observation vector. This method overcomes a disadvantage that the traditional method depends on the fixed threshold value or static weight, so it can more effectively restrain outliers and interference.
- The article not only verifies algorithm performance under the fiducial simulation environment, but also establishes a time-varying anomaly model and some scenes of sea trial track, and assesses performances of the way under different interference and maneuvering degrees.

The remainder of this paper is organized as follows. Section 2 introduces the existing theoretical models and methods, along with our research motivation. Section 3 presents the principle of the proposed robust initial alignment method, detailing the procedures and framework for outlier detection in practical applications. Section 4 provides simulation experiments to demonstrate the effectiveness of the proposed method. Section 5 concludes the paper.

## 2. Problem Statement

In a SINS/DVL integrated navigation system, the velocity measurements provided by the DVL can be used to assist the initial alignment of the SINS. First, by applying the chain rule, the attitude matrix $C_{b}^{n}(t)$ is decomposed as [3]:

(1) $$C_{b}^{n}(t)={\left(C_{n(t)}^{n(0)}\right)}^{T}C_{b}^{n}(0)C_{b(t)}^{b(0)}$$

Among them, t denotes the current time, $C_{n(t)}^{n(0)}$ and $C_{b(t)}^{b(0)}$, respectively, represent the attitude matrices of the navigation frame n and the body frame b with respect to the initial epoch. $C_{b}^{n}(0)$ represents the attitude matrix of the body frame b(0) with respect to the navigation frame n(0) at the initial time, which is a constant matrix. The two time-varying attitude matrices, $C_{n(t)}^{n(0)}$ and $C_{b(t)}^{b(0)}$, can be updated through the attitude matrix differential equations:

(2) $${\dot{C}}_{n(t)}^{n(0)}=C_{n(t)}^{n(0)}(\omega_{in}^{n}(t)\;\times \;)$$

(3) $${\dot{C}}_{b(t)}^{b(0)}=C_{b(t)}^{b(0)}(\omega_{ib}^{b}(t)\;\times \;)$$

Once the time-varying attitude matrices $C_{n(t)}^{n(0)}$ and $C_{b(t)}^{b(0)}$ are obtained, the central task of the OBA method is to determine the constant attitude matrix $C_{b}^{n}(0)$.

Based on the specific force equation, the observation vector equation can be derived as follows [29]:

(4) $$C_{b}^{n}(0)\alpha (t_{M})=\beta (t_{M})$$

The observation vectors $\alpha (t_{M})$ and $\beta (t_{M})$ can be expressed as

(5) $$\alpha (t_{M})=C_{b(t_{M})}^{b(0)}v^{b}(t_{M})-C_{b(t_{m})}^{b(0)}v^{b}(t_{m})-\int_{t_{m}}^{t_{M}}C_{b(\tau )}^{b(0)}f^{b}(\tau )d\tau +\int_{t_{m}}^{t_{M}}C_{b(\tau )}^{b(0)}\omega_{ie}^{b}(\tau )\times v^{b}(\tau )d\tau$$

(6) $$\beta (t_{M})=\int_{t_{m}}^{t_{M}}C_{n(\tau )}^{n(0)}g^{n}d\tau$$

In the above equation, $t_{m}$ denotes the lower integration bound, representing the starting time of the current sliding window, and $t_{M}$ denotes the current time, i.e., the upper integration bound. When $t_{M}-t_{m}=L\Delta t_{a}$ (where L is the fixed length of the sliding window), the integration corresponds to the sliding integration within a window of length $L\Delta t_{a}$.

The discretized forms of the observation vectors $\alpha (t_{M})$ and $\beta (t_{M})$ over the sliding window $\left[t_{m},t_{M}\right]$ can be expressed as follows:

(7) $$\alpha (t_{M})\approx C_{b(t)}^{b(0)}v^{b}(t_{M})-C_{b(t_{m})}^{b(0)}v^{b}(t_{m})-\Delta \alpha_{1}(t_{M})+\Delta \alpha_{2}(t_{M})$$

(8) $$\beta (t_{M})=\sum_{K=m}^{M-1}C_{n(t_{k})}^{n(0)}(\Delta tI_{3\times 3}+\frac{\Delta t^{2}}{2}[\omega_{in}^{n(t_{k})}\times ])g^{n}$$

Here, M denotes the time index of the current time, m denotes the time index of the starting point $t_{m}$ of the current sliding window, M−m represents the length of the sliding window, and Δt is the computation interval. $\Delta \alpha_{1}(t_{M})$ corresponds to the discretized expression of the first integral term in Equation (5), while $\Delta \alpha_{2}(t_{M})$ corresponds to the discretized expression of the second integral term.

According to Equations (4)–(8), the attitude-determination method can uniquely determine the direction cosine matrix $C_{b}^{n}(0)$, thereby achieving moving-base alignment. Moreover, the reference vector $\beta (t_{M})$ is updated from $C_{n(t_{k})}^{n(0)}$ and $g^{n}$, making it less susceptible to sensor output disturbances, whereas the observation vector $\alpha (t_{M})$ is formed directly from the outputs of the IMU and DVL. As a velocity-aiding sensor, the DVL is highly vulnerable to disturbances in complex underwater environments, such as turbulence or multipath effects, which often introduce outliers. These outliers severely degrade the construction accuracy of the vectors $\alpha (t_{M})$ and $\beta (t_{M})$, thus reducing initial alignment precision. In practical applications, DVL data may introduce abnormal observations due to environmental variations, which can cause deviations in the residuals and consequently affect the overall alignment accuracy. Existing robust alignment methods have not sufficiently accounted for the uncertainty in the statistical characteristics of observation data, leading to misclassification and improper handling of outliers, which in turn undermines the stability of the alignment results. Motivated by this, a robust initial alignment is presented in Section 3, where a new outlier-detection criterion based on Mahalanobis distances is established to dynamically detect outliers and adaptively adjust the weights of the observation vectors.

## 3. An Initial Alignment Method with Interference Suppression Based on Mahalanobis Distance

To address above problems, this paper proposes a novel robust initial alignment method. The Mahalanobis distance, which accounts for both correlations among features and scale differences in the data, serves as an effective measure of deviation between outliers and the sample mean. Therefore, it is often used as an indicator for detecting outliers. To detect whether the observation vector in the optimized alignment method is affected by outliers from DVL outputs, we construct the residual vector $h_{i}$ as the data vector in the Mahalanobis distance based on Equation (4).

(9) $$h_{i}=||\beta_{M}|{|}^{2}-||\alpha_{M}|{|}^{2}$$

where the observation vectors $\alpha (t_{M})$ and $\beta (t_{M})$ are abbreviated as $\alpha_{M}$ and $\beta_{M}$, respectively. Let $h_{i}$ denote the residual vector at observation index *i* (*i*, …, *M*), where *M* represents the current time sequence. Therefore, the squared Mahalanobis distance based on $h_{i}$ can be expressed as:

(10) $${D_{M}}^{2}={\left(h_{M}-\mu \right)}^{T}\Sigma^{-1}(h_{M}-\mu )$$

where

(11) $$\mu =\frac{1}{M-1}\sum_{i=1}^{M-1}h_{i}$$

(12) $$\Sigma =\frac{1}{M-1}\sum_{i=1}^{M-1}(h_{i}-\mu ){\left(h_{i}-\mu \right)}^{T}$$

In the above equation, μ denotes the mean of the residual vector $h_{i}$, and Σ represents its covariance matrix. Under the influence of complex marine environment, the statistical characteristics of sensor errors may change. Directly using the Mahalanobis distance to establish an outlier criterion may led to both false alarms and missed detections. Therefore, this paper constructs an outlier criterion based on the cumulative statistical properties of the Mahalanobis distance, as follows:

(13) $$D_{M}>\mu_{D}+2\sigma_{D}$$

where

(14) $$\mu_{D}=\frac{1}{M}\sum_{i=1}^{M}D_{i}$$

(15) $$\sigma_{D}=\sqrt{\frac{1}{M-1}\sum_{i=1}^{M}{\left(D_{i}-\mu_{D}\right)}^{2}}$$

If the Mahalanobis distance $D_{M}$ of the residual vector $h_{i}$ at the current time satisfies Equation (13), the observation vector at that time is regarded as containing an outlier. The corresponding residual vector $h_{i}$ is then excluded from the updates of Equations (11) and (12), thereby preventing the statistics μ and Σ from being contaminated.

Based on the outlier criterion defined in Equation (13), we develop a flexible weighting-update scheme for the observation vector, as follows:

(16) $$w_{M}=\left\{\begin{array}{ll} e^{-{\left(\frac{D_{M}}{\delta }\right)}^{\gamma }} & D_{M}>\mu_{D}+2\sigma_{D}\\ 1 & D_{M}\leq \mu_{D}+2\sigma_{D} \end{array}\right.$$

Here, γ denotes the exponential control parameter. Experimental results show that the optimum performance is achieved when γ=4. The parameter δ acts as the scale factor that controls the mapping from the residual magnitude to the weight attenuation. To ensure consistency with the statistical criterion of the Mahalanobis distance, δ is set to $\sqrt{5.0239}$ in this study, corresponding to the critical value of the chi-square distribution with one degree of freedom n=1 for a 97.5% confidence level.

Based on the outlier criterion in Equation (13), the weighting coefficient $w_{M}$ obtained from Equation (16) is applied to the construction of the attitude observation matrix $K_{M}$. By assigning each innovation term a weight of $w_{M}$, the robustness of the optimized alignment model against abnormal observations can be further enhanced. The updated formula of the observation matrix is therefore given as:

(17) $$K_{M}=K_{M-1}+w_{M}\cdot {\left([\overset{+}{\beta_{M}(t_{M})}]-[\overset{-}{\alpha_{M}(t_{M})}]\right)}^{T}([\overset{+}{\beta_{M}(t_{M})}]-[\overset{-}{\alpha_{M}(t_{M})}])$$

**Figure 1:**
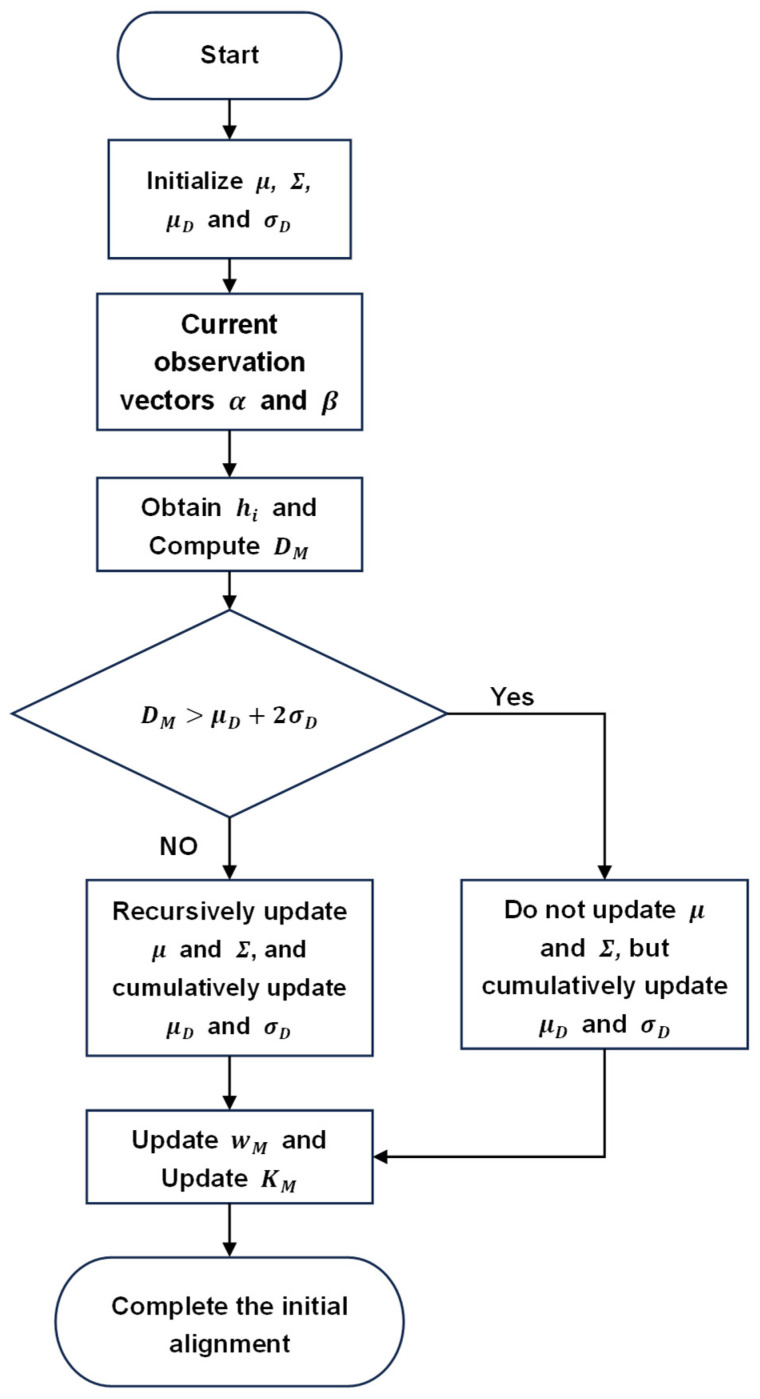
The updating process of the proposed robust initial alignment method.

Based on Equations (4) and (7)–(17), a robust initial alignment method with interference suppression, utilizing the Mahalanobis distance with the proposed outlier-detection criterion, is developed. The specific updating process is illustrated in Figure 1, and the detailed procedure is given as follows:

a. Initialize μ to 0 and Σ to $\sigma_{v}$, and set $\mu_{D}=0$, $\sigma_{D}=\delta$.

b. The observation vectors $\alpha (t_{M})$ and $\beta (t_{M})$ at the current time is calculated by using Equations (7) and (8).

c. Compute the residual vector $h_{i}$ by substituting $\alpha (t_{M})$ and $\beta (t_{M})$ into Equation (9), and then determine the Mahalanobis distance $D_{M}$ at the current time via Equation (10).

d. Determine the presence of outlier interference using Equation (13). If not detected, update μ and Σ recursively with the current residual vector $h_{M}$ and Mahalanobis distance $D_{M}$ by using (11) and (12); If detected, keep μ and Σ unchanged for the next time.

e. Update $\mu_{D}$ and $\sigma_{D}$ by using (14) and (15).

f. Substitute $D_{M}$ into Equation (16) to obtain the updated weight $w_{M}$. Then, use $w_{M}$ in Equation (17) to update the observation matrix $K_{M}$.

g. Finally, the attitude determination algorithm is subsequently employed to complete the in-motion initial alignment.

To enhance computational efficiency, Equations (11) and (12) can be computed using a recursive updating scheme, with the complete procedure provided in Appendix A.

## 4. Experimental Validation and Performance Analysis

### 4.1. Simulation Experiments

**Figure 2:**
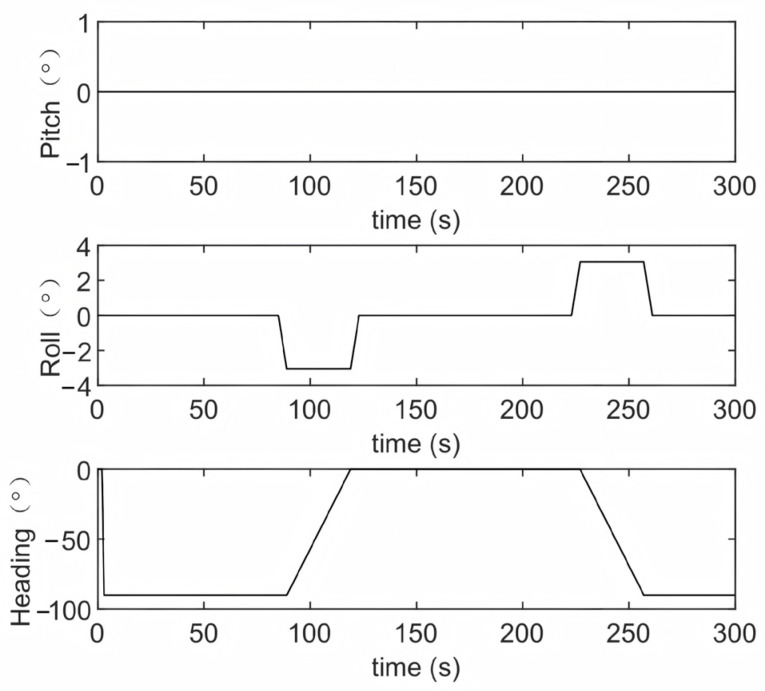
Attitude angle variation curve.

**Figure 3:**
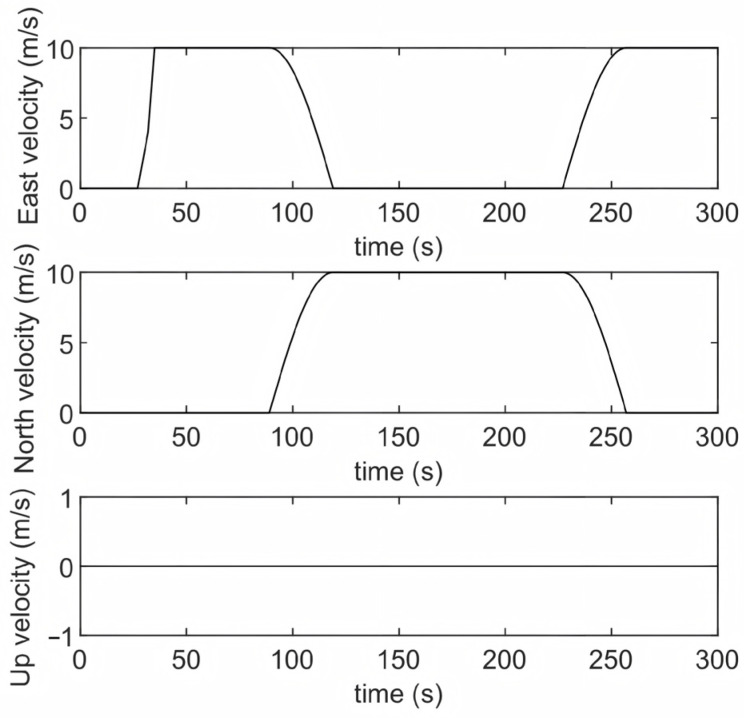
Velocity variation curve.

**Figure 4:**
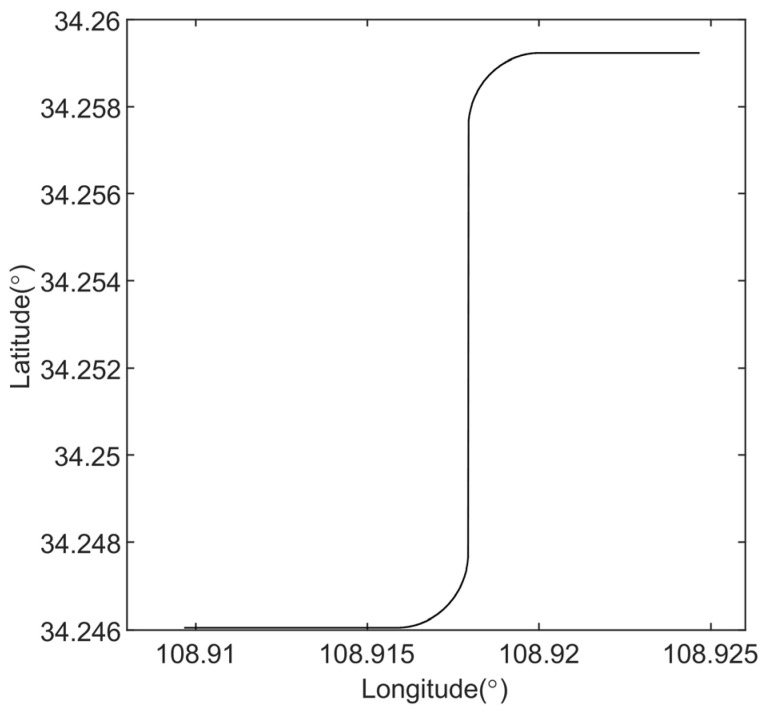
Trajectory variation curve.

To validate the correctness and effectiveness of the proposed robust alignment method, simulation experiments were carried out. A 300-s navigation trajectory was designed under moving-base conditions, with attitude variations, velocity profiles, and the ground-truth trajectory illustrated in Figure 2, Figure 3 and Figure 4. As shown, the trajectory encompasses acceleration, deceleration, turning, and steady linear motion, which are representative of the typical navigation patterns of an underwater vehicle. The sensors’ accuracy configuration is shown in Table 1, where the IMU (100 Hz, gyroscope bias of 0.01°/h, accelerometer bias of 100 μg) and the DVL (5 Hz, velocity measurement noise standard deviation of 0.05 m/s) are specified. Based on the above conditions, we conducted simulations for two sets of outliers probability models separately. The first set simulated the situation where the outliers probability remained constant, while the second set corresponded to the case where the outliers probability changed.

#### 4.1.1. The First Simulation

To model occasional abnormal DVL velocity measurements, outliers are generated by amplifying the instantaneous noise sample by a factor of 200 with a probability of 2%. The DVL measurement error is modeled as

(18) $$\nu_{k1}∼\left\{\begin{array}{lll} N(0,\Sigma_{\nu }), & w.p. & 1-p_{0}\\ N(0,\alpha_{0}^{2}\Sigma_{\nu }), & w.p. & p_{0} \end{array}\right.$$

where $\nu_{k1}$ is the DVL velocity measurement error at the k1-th moment, $\Sigma_{\nu }$ is the nominal DVL noise covariance, $p_{0}=0.02$ is the fixed outlier probability, and $\alpha_{0}=200$ is the noise amplification factor.

**Figure 5:**
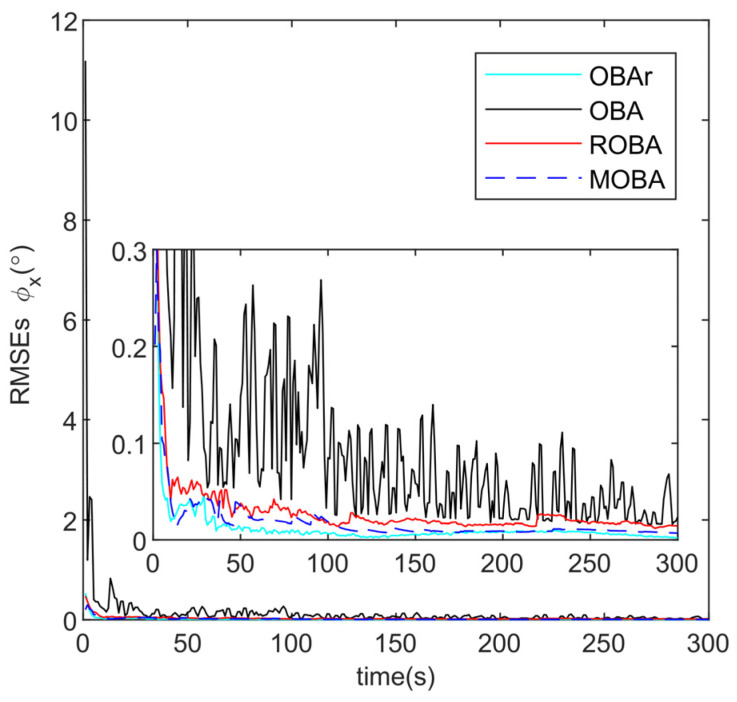
Roll angle error curve.

**Figure 6:**
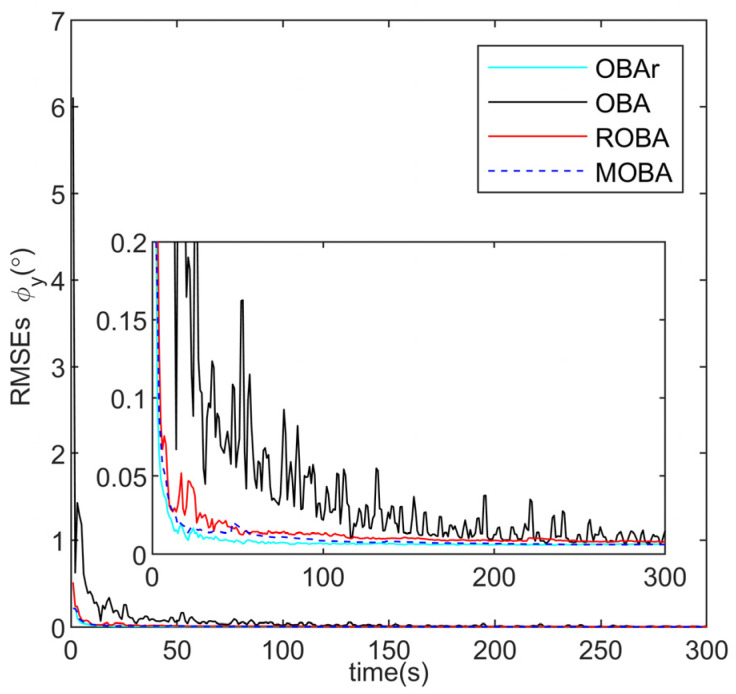
Pitch angle error curve.

**Figure 7:**
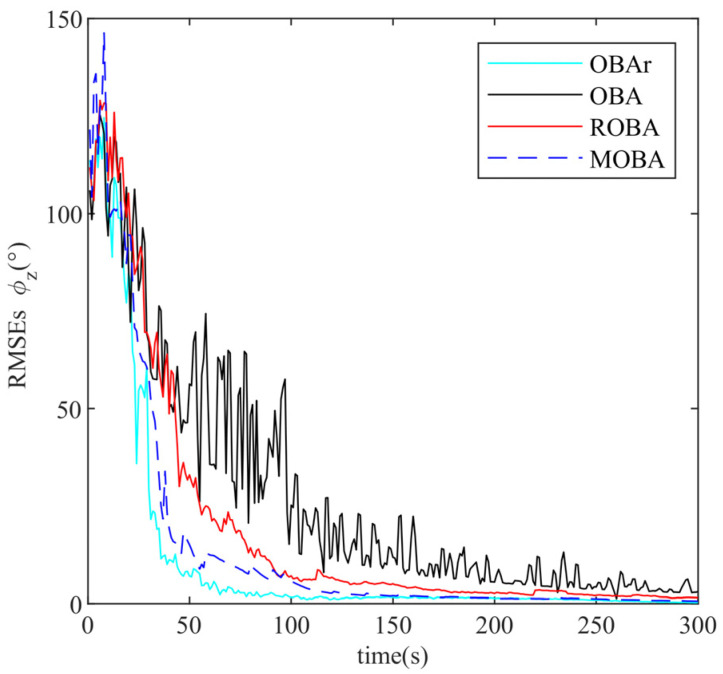
Heading angle error curve.

The proposed method (MOBA) is compared with several alternatives, including the conventional optimization-based alignment (OBA) [30], the OBA method without additional outlier injection (OBAr), and the robust OBA method (ROBA) [25]. The root-mean-square errors (RMSE) of alignment obtained from 10 independent runs are plotted in Figure 5, Figure 6 and Figure 7 and summarized in Table 2.

As illustrated in Figure 5 and Figure 6, the pitch and roll angles are only marginally influenced by outliers, and all methods achieve rapid convergence within 0.1° in less than 50 s. Nevertheless, the alignment results obtained by the MOBA method are closer to those of the ideal OBAr. Figure 7 and Table 2 further demonstrate that the OBA method yields larger alignment errors, whereas both ROBA and MOBA exhibit robustness against outlier interference, with MOBA delivering superior alignment accuracy. Notably, the proposed MOBA method is the only one that maintains heading accuracy within the acceptable range of 1°, this is the standard benchmark in simulation and general underwater navigation evaluations, while both OBA and ROBA fall outside this range. This improvement arises from the fact that OBA lacks any mechanism to resist interference, while ROBA relies solely on empirically selected threshold parameters for suppression. By contrast, MOBA is capable of identifying outliers through a statistical detection criterion and dynamically updating the monitoring statistics. In addition, it incorporates an adaptive weighting factor to regulate the influence of observational innovations on the alignment process, thereby ensuring enhanced robust alignment performance.

#### 4.1.2. The Second Simulation

To further assess the behavior of the alignment algorithms under more diverse and time-varying disturbance conditions, we conducted an additional simulation experiment using the segmented outlier model. The 300-s experiment was divided into five 60-s phase. In each phase, the DVL measurement noise follows the same nominal distribution, while sporadic outliers are generated with phase-dependent probabilities and magnitudes. Specifically, the DVL velocity measurement error $\nu_{k2}$ in phase s is modeled as

(19) $$\nu_{k2}∼\left\{\begin{array}{ll} N(0,\Sigma_{\nu }), & w.p.\;1-p_{s}\\ N(0,\alpha_{s}^{2}\Sigma_{\nu }), & w.p.\;p_{s} \end{array}\right.$$

where k2(k2=1,2,…) denotes each discrete time sample, and s(s=1,2,…,5) indicates the specific 60-s time segment to which the current sample k2 belongs. $p_{s}$ is the outlier probability in segment $s(p_{s}\leq 0.02)$, $\alpha_{s}$ is the noise amplification factor $\left(\alpha_{s}\leq 200\right)$, consistent with the implementation in which outliers are produced by scaling the instantaneous noise sample by $\alpha_{s}$. The segment-specific parameters are listed in Table 3. All other simulation settings—including the attitude maneuver profile, velocity inputs, and reference trajectory—were kept strictly identical to those used in the first simulation to ensure fair comparison.

**Figure 8:**
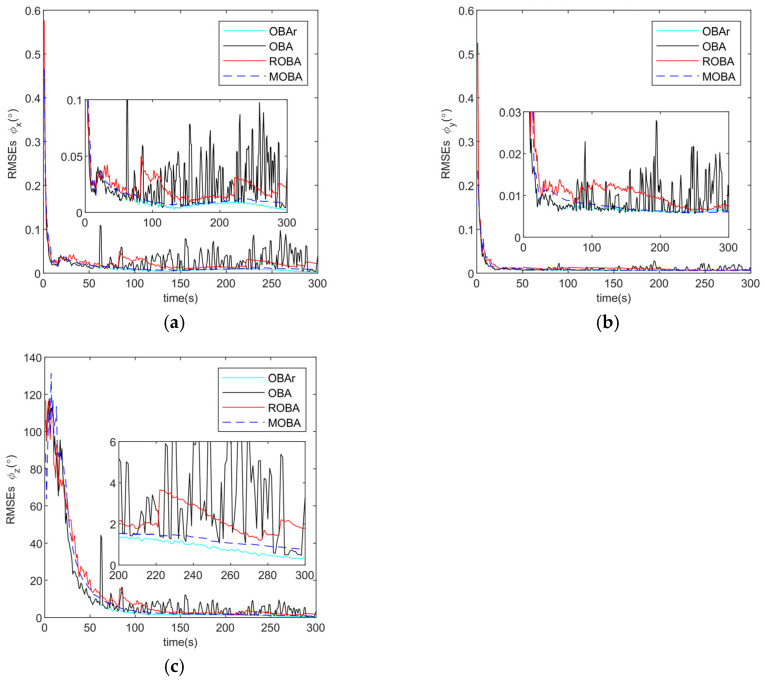
(**a**) Roll angle error curve; (**b**) Pitch angle error curve; (**c**) Heading angle error curve;.

The alignment results of the roll, pitch, and heading angles for the five-segment disturbance model are displayed in Figure 8a–c, and the corresponding RMSE values during the final 50 s are summarized in Table 4. It can be observed that the proposed MOBA method consistently maintains the highest robustness across all disturbance phases, and is the only method that keeps the heading accuracy within the commonly accepted practical tolerance of approximately 1° under these more challenging conditions.

### 4.2. Field Test

**Figure 9:**
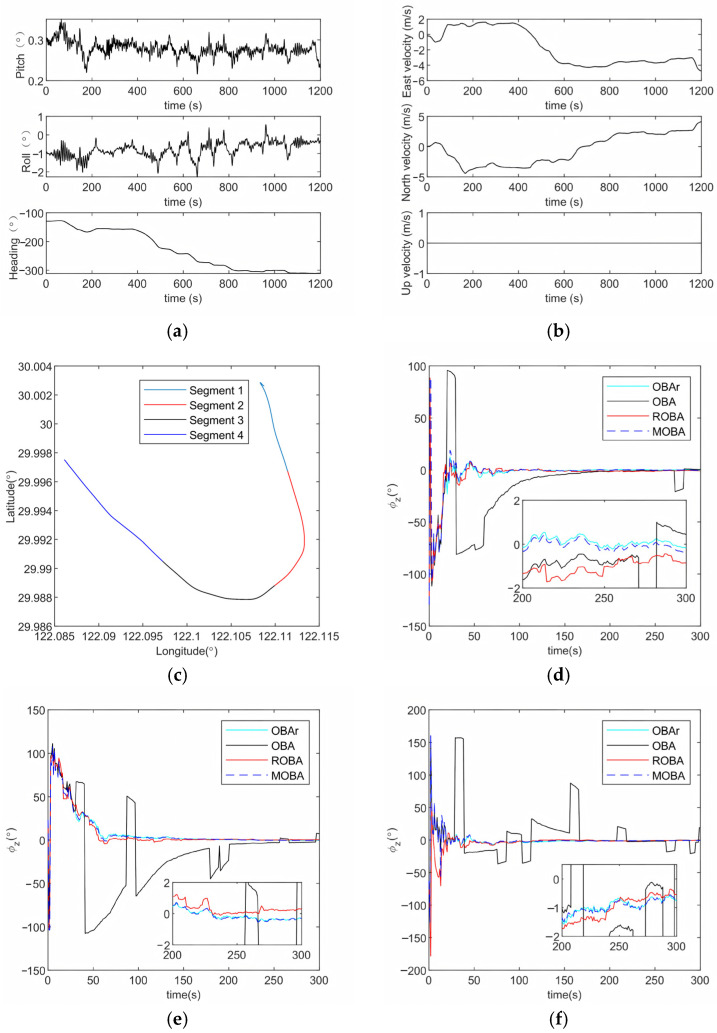

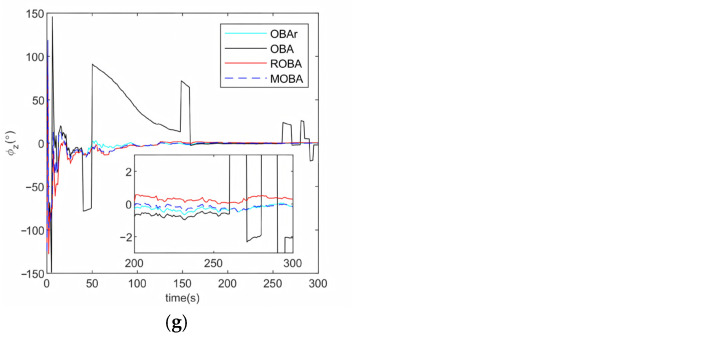
(**a**) Attitude angle variation curve. (**b**) Velocity variation curve. (**c**) Trajectory variation curve. (**d**) Heading angle error for Segment 1. (**e**) Heading angle error for Segment 2. (**f**) Heading angle error for Segment 3. (**g**) Heading angle error for Segment 4.

To verify the actual performance of the proposed MOBA method, we conducted an initial alignment experiment using a 1200-s sea trial dataset. The attitude, velocity, and motion trajectory within the complete period are shown in Figure 9a–c. To facilitate performance evaluation, we divided the entire dataset into four consecutive 300-s segments, and conducted independent initial alignment tests for each period to examine the robustness of the method under different motion patterns. The experimental data were collected using a fiber-optic strapdown inertial navigation system (FSINS), a DVL, and a high-precision integrated navigation system, with the detailed configuration summarized in Table 5. The FSINS consisted of a fiber-optic gyroscope (bias stability: 0.01°/h, angle random walk: $<0.03{}^{\circ}/h/\sqrt{Hz}$) and accelerometers (bias stability: 100 μg, velocity random walk: $<10\mu g/\sqrt{\mathrm{Hz}}$), with an output rate of 98 Hz. The DVL provided measurements at 1 Hz with a velocity measurement noise standard deviation of 0.1 m/s. The outputs of the high-precision integrated navigation system were used as reference data. Outliers were randomly injected into the DVL output with a 2% probability to emulate occasional abnormal observations in practical systems and to evaluate the algorithm’s robustness under non-ideal conditions. Figure 9d–g present the heading estimation errors of the four methods (OBAr, OBA, ROBA, and MOBA) for the four trajectory segments, while Table 6 summarizes the corresponding RMSE values computed over the final 50 s of each alignment. The simulation and practical test results show that, under outlier disturbances, all compared methods exhibit very similar RMSE performance in roll and pitch. Therefore, only the accuracy comparison of the heading angle is presented here.

The results in Figure 9d–g indicate that, in terms of heading angle accuracy, the OBA method fails to meet the commonly accepted practical accuracy requirement of approximately 1°. This is because the OBA method lacks any mechanism to suppress DVL outlier disturbances, making its heading estimation highly sensitive to outlier contamination. In contrast, both the ROBA method and the proposed MOBA method are able to keep the heading angle RMSE within 1°. Among them, MOBA exhibits more stable and consistently reliable performance across all four trajectory segments and remains unaffected even under higher maneuver intensity or increased outlier disturbances. This demonstrates that MOBA can maintain strong engineering applicability across a wider range of operating scenarios.

## 5. Conclusions

In this paper, a method based on outlier-Resistant Initial Alignment of DVL-Aided SINS Using Mahalanobis Distance was proposed. By performing dynamic outlier detection together with adaptive weighting of the observations, the method effectively suppresses the influence of outlier disturbances during the alignment process. Experimental results show that, compared with existing approaches, the proposed MOBA method achieves higher alignment accuracy and stronger robustness in the presence of DVL outliers, demonstrating its suitability for moving-base alignment of DVL-aided SINS in complex marine environments. The method also has limitations, it is currently applicable only to medium- and high-precision initial alignment. Similar to most optimization-based initial alignment schemes, its performance degrades under low-precision sensor conditions, where moving-base alignment remains a challenging problem. Future work will consider incorporating error estimation and compensation mechanisms to improve alignment performance in low-precision scenarios.

## Figures and Tables

**Table 1:** Sensor configuration.

Sensor	Parameter	Value
IMU	Sampling frequency	100 Hz
	Gyroscope bias	0.01°/h
	Gyroscope noise	$0.001{}^{\circ}/\sqrt{h}$
	Accelerometer bias	100 μg
	Accelerometer noise	$10\;\mu g/\sqrt{\mathrm{Hz}}$
DVL	Sampling frequency	5 Hz
	Velocity noise standard deviation	0.05 m/s
	Outlier probability	random noise amplified ×200 2%

**Table 2:** Comparison of the RMSE of the attitude angles in the first simulation.

Error Type	OBAr	OBA	ROBA	MOBA
Pitch	0.005°	0.04°	0.017°	0.009°
Roll	0.006°	0.011°	0.008°	0.006°
Heading	0.57°	4.39°	1.85°	0.89°

**Table 3:** Parameters of the time-segmented outlier model.

Segment	Duration (s)	Probability p	Magnitude mag
1	0–60	0.001	10
2	60–120	0.010	50
3	120–180	0.015	100
4	180–240	0.020	150
5	240–300	0.020	200

**Table 4:** Comparison of the RMSE of the attitude angles in the second simulation.

Error Type	OBAr	OBA	ROBA	MOBA
Pitch	0.005°	0.04°	0.02°	0.001°
Roll	0.006°	0.012°	0.007°	0.011°
Heading	0.64°	4.36°	1.79°	0.92°

**Table 5:** Sensor configuration.

Sensor	Parameter	Value
IMU	Sampling frequency	98 Hz
	Gyroscope bias stability	0.01°/h
	Gyroscope angle random walk	$<0.03{}^{\circ}/h/\sqrt{\mathrm{Hz}}$
	Accelerometer bias stability	100 μg
	Accelerometer velocity random walk	$<10\mu g/\sqrt{\mathrm{Hz}}$
DVL	Sampling frequency	1 Hz
	Standard deviation of velocity measurement noise	0.1 m/s
	Outlier probability	probability of 2%

**Table 6:** Heading RMSE for Segment 1–4.

Segment	OBAr	OBA	ROBA	MOBA
1	0.1160°	8.7566°	0.7660°	0.2001°
2	0.3416°	3.2290°	0.2102°	0.3725°
3	0. 8062°	11.1331°	0.6311°	0.8092°
4	0.2623°	13.7160°	0.3188°	0.2727°

## Data Availability

The original contributions presented in this study are included in the article. Further inquiries can be directed to the corresponding author.

## Nomenclature

Notations	Definitions
b	Body frame
ⅇ	Earth frame
ⅈ	Inertial frame
n	Navigation frame (geographic frame)
$f^{b}$	Specific force in b-frame
$g^{n}$	Gravity vector
$\omega_{ib}^{b}$	Body angular rate in b frame
$v^{b}$	Ground velocity of SINS in b frame
$C_{x_{1}}^{x_{2}}$	Attitude matrix from $x_{1}$-frame to $x_{2}$-frame
$C_{b}^{n}(t)$	Attitude matrix from body frame b to navigation frame n at time t
x(t)	Value of the variable x at time t
$\omega_{x_{1}x_{2}}^{x_{3}}$	Angular velocity of $x_{2}$-frame with respect to $x_{1}$-frame projected in $x_{3}$-frame
$\omega_{in}^{n}\times$	Skew-symmetric matrix of the angular velocity of the inertial frame with respect to the navigation frame, projected in n-frame, i.e., the cross-product operator satisfying $(\omega_{in}^{n}\times )x=\omega_{in}^{n}\times x.$
$\omega_{ib}^{b}\times$	Skew-symmetric matrix of the angular velocity of the inertial frame with respect to the body frame, expressed in the b-frame, i.e., the cross-product operator satisfying $(\omega_{ib}^{b}\times )x=\omega_{ib}^{b}\times x$ for any vector x.

## Appendix A

Let the current discrete time be denoted as M, and let $n_{M}$ represent the total number of non-outlying observations from the initial time step up to time M. Let $\mu_{M}$ denote the sample mean vector calculated from the first $n_{M}$ data points, and $\Sigma_{M}$ the corresponding sample covariance matrix. When the observation vector at the current time step is identified as a non-outlying data point, the Mahalanobis distance can be updated recursively using the following procedure:

Update the number of data points

(A1) $$n_{M}=n_{M-1}+1$$

Update the mean $\mu_{M}$

(A2) $$\mu_{M}=\mu_{M-1}+\frac{1}{n_{M}}(h_{M}-\mu_{M-1})$$

Update the covariance matrix

(A3) $$\Sigma_{M}=\frac{n_{M-1}}{n_{M}}\Sigma_{M-1}+\frac{1}{n_{M}}((h_{M}-\mu_{M-1}){\left(h_{M}-\mu_{M-1}\right)}^{T})-\frac{n_{M-1}}{n_{M}}(h_{M}-\mu_{M-1}){\left(h_{M}-\mu_{M-1}\right)}^{T}$$

By employing the above expression, and under the condition of no outlier observations, the mean and covariance can be updated using the recursive formulas.

When the observation vector at the current time step is affected by outliers:

Update the number of data points

(A4) $$n_{M}=n_{M-1}$$

Update the mean $\mu_{M}$

(A5) $$\mu_{M}=\mu_{M-1}$$

Update the covariance matrix

(A6) $$\Sigma_{M}=\Sigma_{M-1}$$

By employing the above expression, when outlier observations are present, the mean and variance are retained from the previous time step to prevent contamination by the outliers and to avoid compromising the judgment at the next time step.
